# Supplementary material for: Quantifying contributions of chlorofluorocarbon banks to emissions and impacts on the ozone layer and climate
Source: Nat Commun. 2020 Mar 17;11:1380. doi: 10.1038/s41467-020-15162-7 (PMC7078219; doi:10.1038/s41467-020-15162-7)
Supplement: Supplementary file 1 — Supplementary Information [file 41467_2020_15162_MOESM1_ESM.pdf]

Supplementary Information

Quantifying contributions of chlorofluorocarbon banks to emissions and impacts on the ozone layer and climate

Lickley et al.

Supplementary Methods 1

Direct Emissions and Release Fraction

We develop joint sample time series for direct emissions (DE), and release fractions (RF) using parameters to represent 1) how production is apportioned across each type of equipment, and 2) the timing of chlorofluorocarbon loss by equipment type for each year. We describe our priors for each of these parameters below, followed by a description of the sampling procedure.

Parameter 1: Proportion of production by equipment type

Estimates for the distribution of production across equipment type are constructed using AFEAS production data (https:// unfccc.int). We assume that this AFEAS production data provides a basis for how production is apportioned across equipment type. The equipment type categories for CFC-11, CFC-12, and CFC-113 are shown in Tables S1, S2, and S3. We use annual production data from the years 1930 to 2000. For each year, we construct a prior distribution for each equipment type, k, as we do for total production, described above. That is, if $\mathrm{Prod}_{0,k,t}$ is the reported production for equipment type, k, in year, t, then we assume the prior distribution for production follows a lognormal distribution such that $\mathrm{Prod}_{k,t} \sim \mathrm{Prod}_{0,k,t} \times (0.1 * \mathrm{Log}(0,0.5) + 0.95)$. For each sampled time series, we sample independently from the respective distribution for each equipment type and each year. After AFEAS data ends in 2000, we use that year for the priors in later years. The one exception is for the CFC-11 unexpected emissions scenario. Because we have no knowledge about the type of production for this additional emission scenario, for each sample time series we randomly partition production across equipment type, assuming each type to be equally probable.

Parameter 2: Emission function by equipment type

Estimates for the emission function by equipment type are based on values from Ashford (2004). We construct prior distributions for each emission function related to the equipment types as given in Tables S1 - S3. Broadly, for equipment types with a year 1 emission rate equal to half of production, we chose priors with beta distributions and a standard deviation of 20% of year 1's emission rate. For slower release functions, where annual release fractions are a low percentage of the bank, we use a lognormal distribution for the prior with standard deviations of 15%-20% of the release rate. For emissions functions defined using a lifetime, we assume a lognormal distribution around the lifetime with a 20% uncertainty.

Constructing sampled time series

For Bayesian Parameter Estimation, we sample a total of 500,000 time series. For each joint sample of DE and RF, our sampling procedure is as follows:

1. For each year, t, and each equipment type, k, sample a production value $\mathrm{Prod}_{k,t}$ from its prior distribution.
2. For each equipment type, k, sample an emission function for year 1's release rate $\mathrm{EF}_{k,\mathrm{y1},}$, and for the banks release rate $\mathrm{EF}_{k,\mathrm{bank}}$ from their prior distributions. We use the term $EF_{k,\mathrm{bank}}$ to refer to the release fraction specific to equipment type and RF to refer to the release fraction of a specific gas, which aggregates across

all types of equipment for that gas. $EF_{k,y1}$ is the RF in the year of production, which we term Direct Emissions (DE) when aggregated across all equipment types.

3. For each equipment type, k, use the samples from Step 1 and Step 2 and simulate forward in time to create a sample time series for the bank ($\mathrm{Bank}_{k,t}$), absolute direct emissions ($\mathrm{DirectEmiss}_{k,t}$), and the bank's emissions ($\mathrm{BankEmiss}_{k,t}$), as follows:

$$\mathrm{Bank}_{k,t} = \left(1 - \mathrm{EF}_{\mathrm{bank},k}\right) \times \mathrm{Bank}_{k,t-1} + \left(1 - \mathrm{EF}_{k,y1}\right) \times \mathrm{Prod}_{k,t}$$

$$\mathrm{DirectEmiss}_{k,t} = \mathrm{EF}_{k,y1} \times \mathrm{Prod}_{k,t}$$

$$\mathrm{BankEmiss}_{k,t} = \mathrm{EF}_{\mathrm{bank},k} \times \mathrm{Bank}_{k,t-1}$$

The initial bank size for 1930 is assumed equal to zero.

4. Estimate total DE and RF by summing each equipment type's bank, absolute direct emissions, and bank emissions as follows:

$$\mathrm{RF}_t = \frac{\sum_k \mathrm{BankEmiss}_{k,t}}{\sum_k \mathrm{Bank}_{k,t-1}}$$

$$\mathrm{DE}_t = \frac{\sum_k \mathrm{DirectEmiss}_{k,t}}{\sum_k \mathrm{Prod}_{k,t}}$$

The resulting time series for DE_t and RF_t represents a sample from the prior distribution used in the Bayesian Parameter Estimate simulation model described in the main text. The time series for CFC-11, 12, and 113, are shown in Supplementary Figures 14-16.

Supplementary Note 1

CFC-113 Emissions and Lifetime Uncertainties

Uncertainties in CFC-113 lifetimes are sometimes estimated from observations of annual concentration changes by making assumptions about emissions (e.g., assumed zero emissions after the international global CFC phaseout date, which would clearly be an incorrect approach if additional emissions are actually occurring). Such an approach was taken to derive a very long upper limit to the CFC-113 lifetime uncertainty of around 109 years in the SPARC lifetime assessment. However, our analysis suggests that additional emissions are much more likely (see main text). We therefore estimate uncertainties in total CFC-113 emissions assuming an uncertainty range of +/- 15% in the CFC-113 lifetime from the SPARC lifetime uncertainty estimate using tracer-tracer correlations (and adopted a best estimate of 85 years). Supplementary Figure 13 shows the result. Recently, positive trends in the CFC-113a isomer[1] of 113 have been reported[1]; those findings cannot account for the non-zero emissions shown below.

Supplementary Tables

Supplementary Table 1: CFC-11parameter categories based on AFEAS data, and emissions functions with values from Ashford (2004). Prior distributions were chosen to reflect our assumption about the uncertainties in estimated values from Ashford (2004). AFEAS data can be found at https://unfccc.int/files/methods/other_methodological_issues/interactions_with_ozone_layer/application/pdf/cfc1100.pdf

Equipment categories	Non-hermetic Refrigeration	Blowing Agents Closed Cell Foam	Open Cell Foam, Aerosols and Others
Year 1:	Year 1: 7% release	NA	Year 1: 83% release for Open Cell Foam, 50% release for Aerosols
Prior Distribution	Lognormal with parameters such that μ= 7% and σ = 3.5%		Beta, parameters 8, 6 which gives: μ = 57%, σ = 13%
Subsequent years:	Life years: 10 years	3.66% of bank	Year 2: 17% release for open cell foam, 50% release for aerosols
Prior Distribution	Lognormal μ = 10 years, σ =2 year	Lognormal with parameters such that μ = 3.66% and σ = 1.83%	Modeled jointly with Year 1 distribution. (1 – Year 1 release)

Supplementary Table 2: CFC-12 parameter categories based on AFEAS data, and emissions functions with values from Ashford (2004). Prior distributions were chosen to reflect our assumptions about the uncertainties in estimated values from Ashford (2004). AFEAS data can be found at https://unfccc.int/files/methods/other_methodological_issues/interactions_with_ozone_layer/application/pdf/cfc1200.pdf

Equipment categories	Non-hermetic Refrigeration	Hermetic Refrigeration	Blowing Agents Closed Cell Foam	Open Cell Foam, Aerosols and Others
Year 1:	Year 1: 7% release	Year 1: 2% release	Year 1: 50% release	Year 1: 83% release for Open Cell Foam, 50% release for Aerosols
Prior Distribution	Lognormal with parameters s.t. μ= 7%, σ = 3.5%	Lognormal with parameters s.t. μ= 2%, σ = 1.4%	Beta Distribution with parameters 12, 12 which gives: μ = 50%, σ = 10%	Beta, parameters 8, 6 which gives: μ = 57%, σ = 13%

Subsequent years:<br><br>Prior Distribution	Life years: 10 years<br><br>Lognormal<br>μ = 10 years, σ =2 year	Life years: 20 years<br><br>Lognormal<br>μ = 20 years, σ =4 year	Year 2: 50% release<br><br>modeled jointly with Year 1.<br>(1 – Year 1 release)	Year 2:17% release for open cell foam, 50% release for aerosols.<br><br>Modeled jointly with Yr 1 (1-Year 1 release)

Supplementary Table 3: CFC-113 parameter categories based on AFEAS data, and emissions functions with values from Ashford (2004). Prior distributions were chosen to reflect our assumptions about the uncertainties in estimated values from Ashford (2004). AFEAS data can be found at https://unfccc.int/files/methods/other_methodological_issues/interactions_with_ozone_layer/application/pdf/cfc1300.pdf

AFEAS Equipment categories (corresponding Ashford (2004) table category)	Short Banking Times (Solvent or Aerosol or closed cell foam)	Long term (Hermetic Refrigeration)
Year 1:<br><br>Prior Distribution	Year 1: 50% release<br><br>Beta Distribution with parameters 12, 12 which gives:<br>μ = 50%, σ = 10%	Year 1: 2% release<br><br>Lognormal with parameters s.t.<br>μ= 2%, σ = 1%
Subsequent years:<br><br>Prior Distribution	Year 2: 50% release<br><br>modeled jointly with Year 1.<br>(1 – Year 1 release)	Life years: 20 years<br><br>Lognormal<br>μ = 20 years, σ =4 year

Supplementary Figures

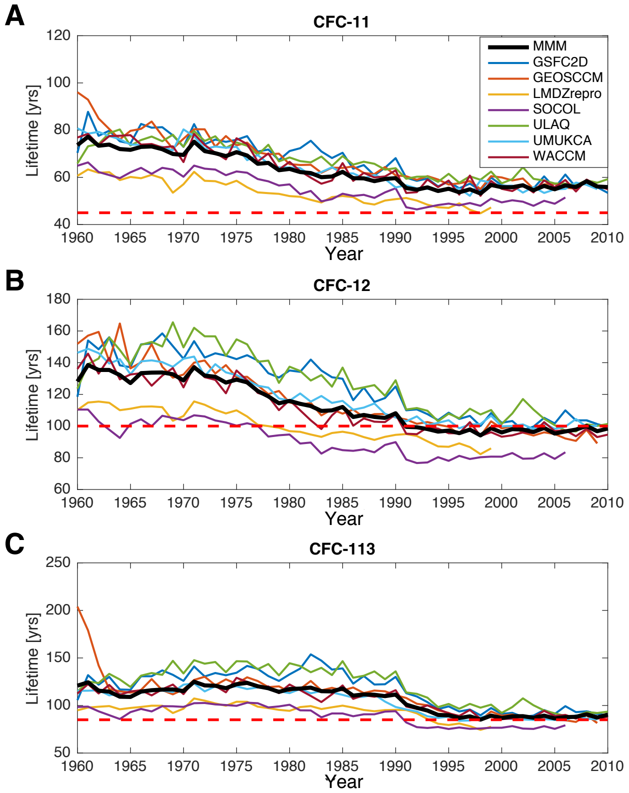

Supplementary Figure 1: Atmospheric lifetimes for (a) CFC-11, (b) CFC-12, and (c) CFC-113 from the SPARC (2013) report. Colored solid lines indicate each model's projected lifetimes. The thick black line is the multi-model mean (MMM), and the dotted red line is the WMO (2003) constant lifetime value.

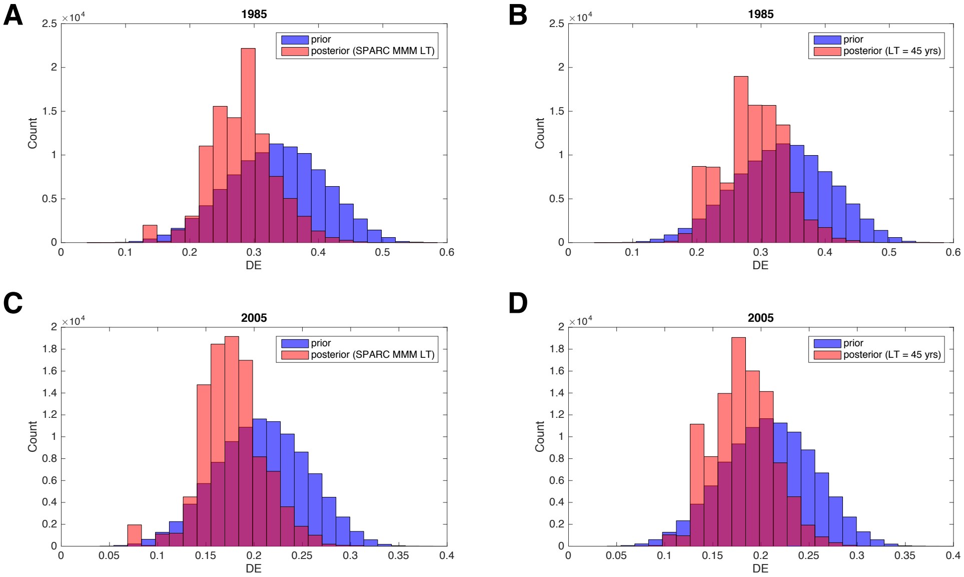

Supplementary Figure 2: CFC-11 samples from prior (blue) and posterior (pink) distributions of direct emissions (DE) for two select years in the time series: 1985 (a, b) and 2005 (c, d). The BPE model is implemented using the SPARC MMM atmospheric lifetime scenario (a, c) and the constant lifetime scenario (b, d) is for a lifetime of 45 years.

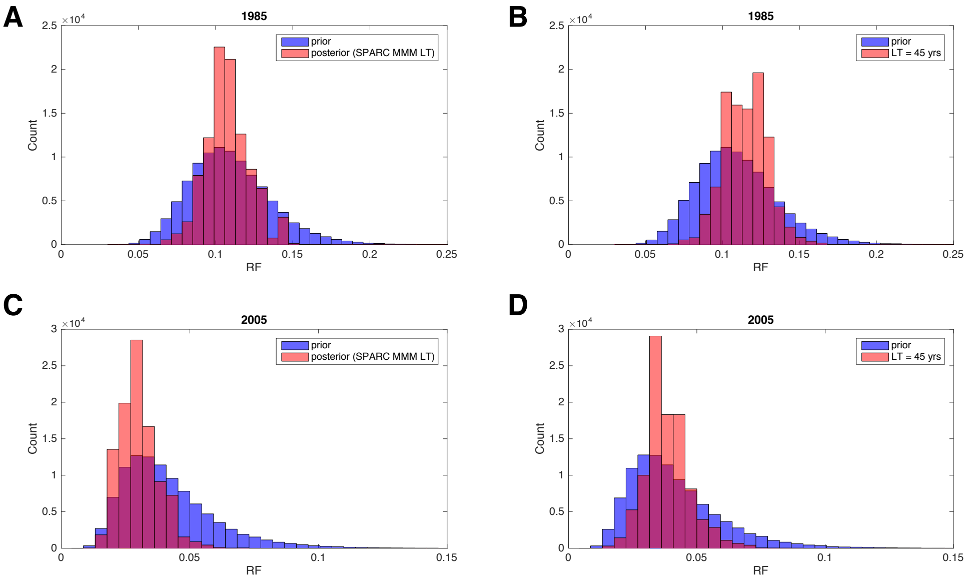

Supplementary Figure 3: CFC-11 samples from prior (blue) and posterior (pink) distributions of release fraction (RF) for two illustrative years in the time series: 1985 (a, b) and 2005 (c, d). The BPE model is implemented using the SPARC MMM atmospheric lifetime scenario (a, c) and the constant lifetime scenario (b, d) is for a lifetime of 45 years.

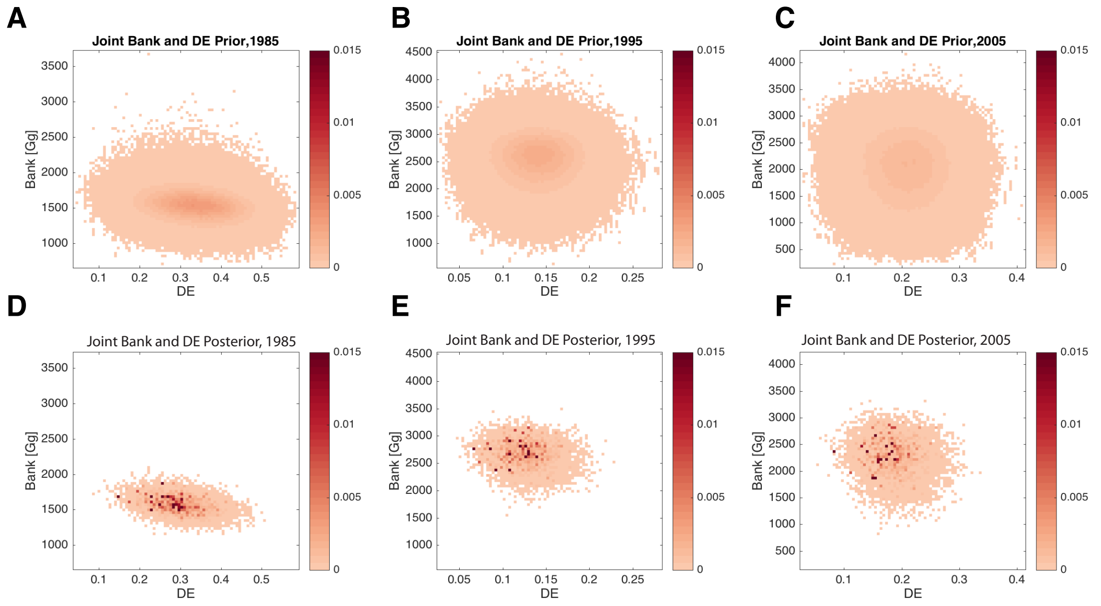

Supplementary Figure 4: CFC-11 Joint DE and bank prior (a – c) and posterior (d – f) distributions for three selected years for the SPARC multi-model mean BPE lifetime run. The color bar indicates the relative sampling frequency.

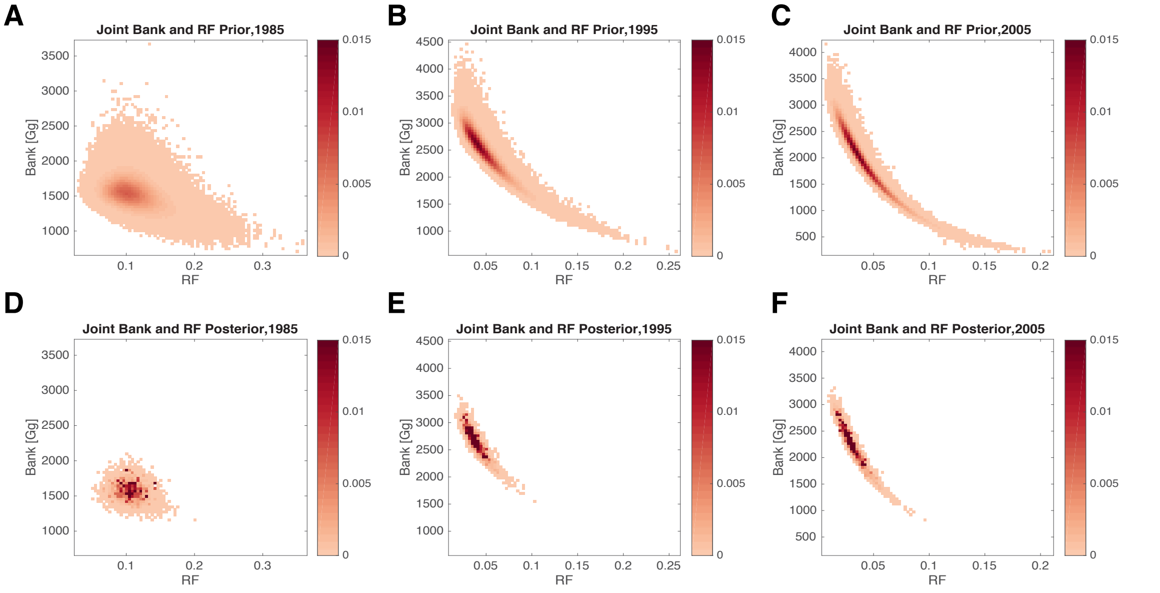

Supplementary Figure 5: CFC-11 Joint RF and bank prior (a – c) and posterior (d – f) distributions for three selected years for the SPARC multi-model mean BPE lifetime run. The color bar indicates the relative sampling frequency.

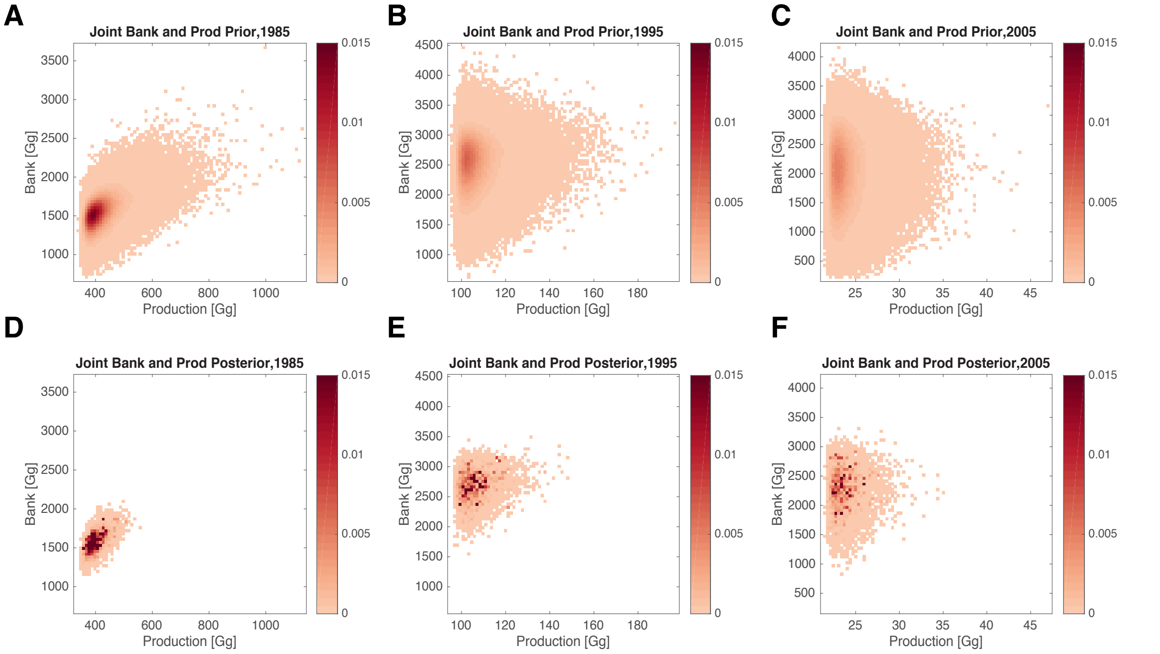

Supplementary Figure 6: CFC-11 Joint production and bank prior (a – c) and posterior (d – f) distributions for three selected years for the SPARC multi-model mean BPE lifetime run. The color bar indicates the relative sampling frequency.

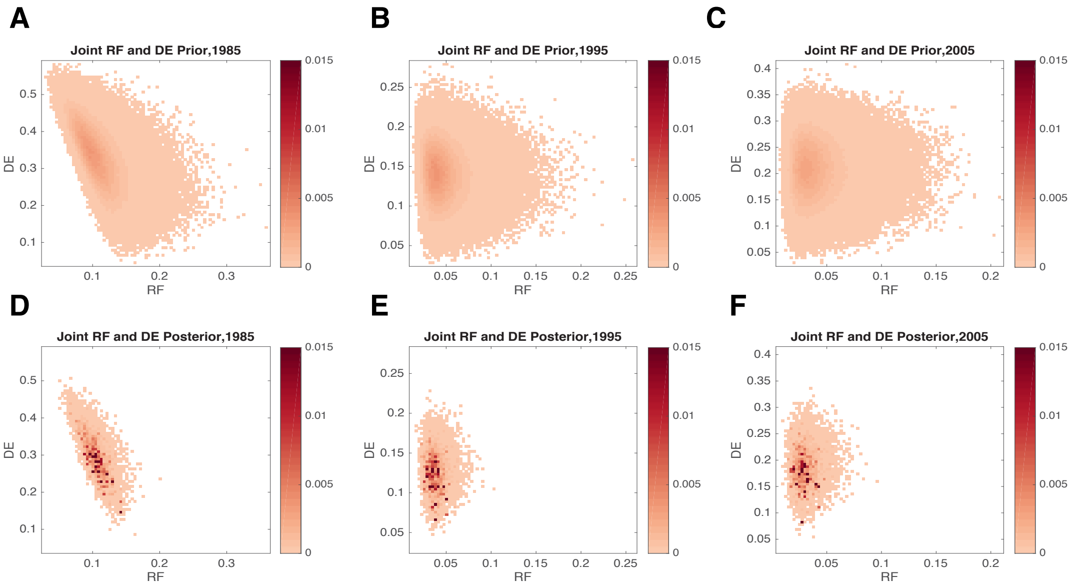

Supplementary Figure 7: CFC-11 Joint DE and RF prior (a – c) and posterior (d – f) distributions for three selected years for the SPARC multi-model mean BPE lifetime run. The color bar indicates the relative sampling frequency.

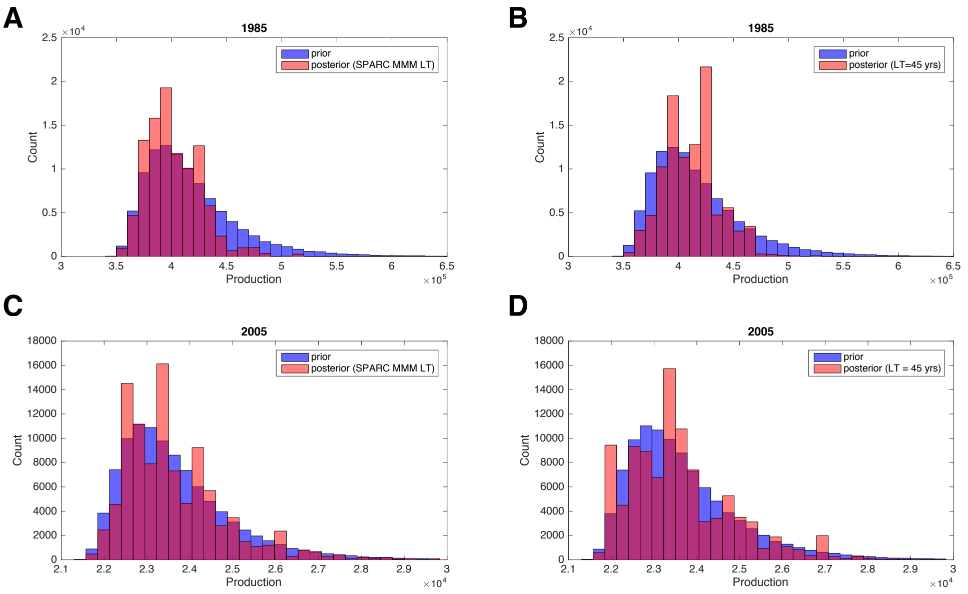

Supplementary Figure 8: CFC-11 samples from prior (blue) and posterior (pink) production distributions for two selected years in the time series: 1985 (a, b) and 2005 (c, d). The BPE model is implemented using the SPARC MMM atmospheric lifetime scenario (a, c) and the constant lifetime scenario (b, d) is for a lifetime of 45 years.

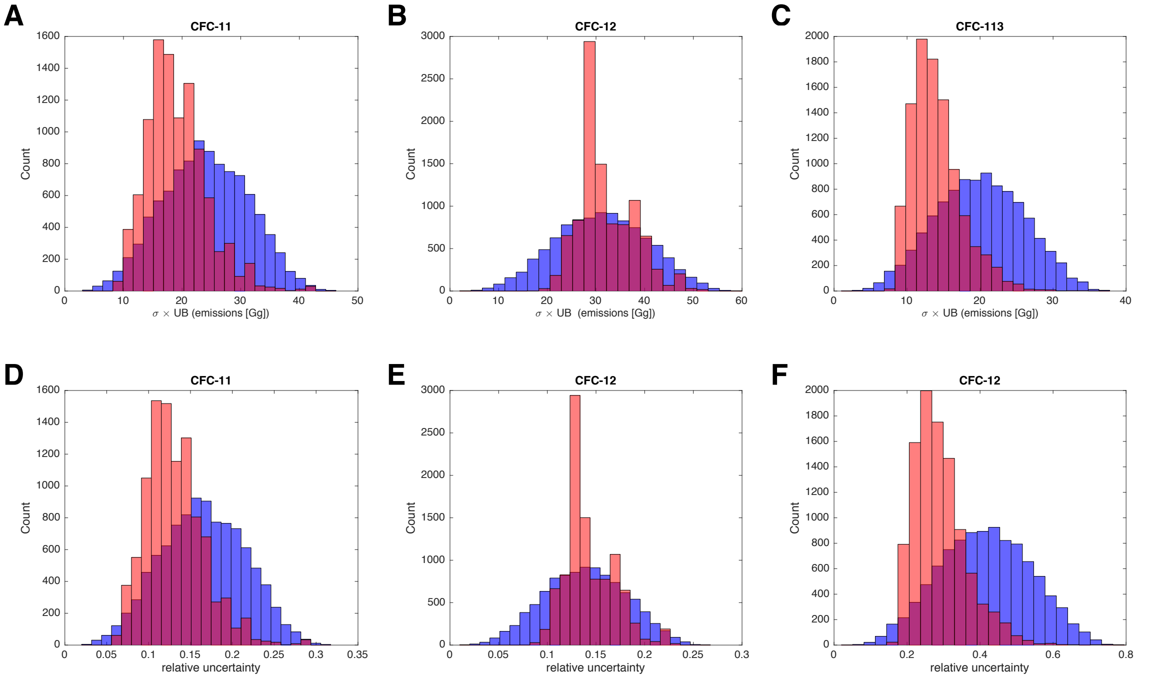

Supplementary Figure 9: (a – c) Prior (blue) and posterior (pink) samples of $\sigma \times UB$, the uncertainty term estimated in the BPE for each of the molecules run using the SPARC MMM LT scenario. (d – f) As in (a – c) except the x-axis represents $\sigma \times UB$ as a fraction of the mean of observationally-derived emissions.

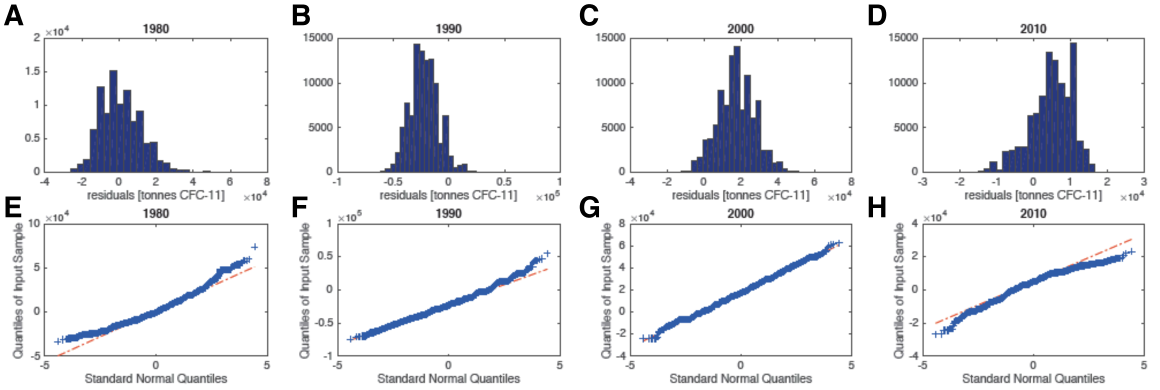

Supplementary Figure 10: (a – d) Histogram of residuals (D_{emiss}-M(θ)$_{emiss}$) for 1985, 1995, and 2005 for the CFC-11 run using the SPARC MMM LT scenario. (e – h) qqplot of residuals for corresponding years.

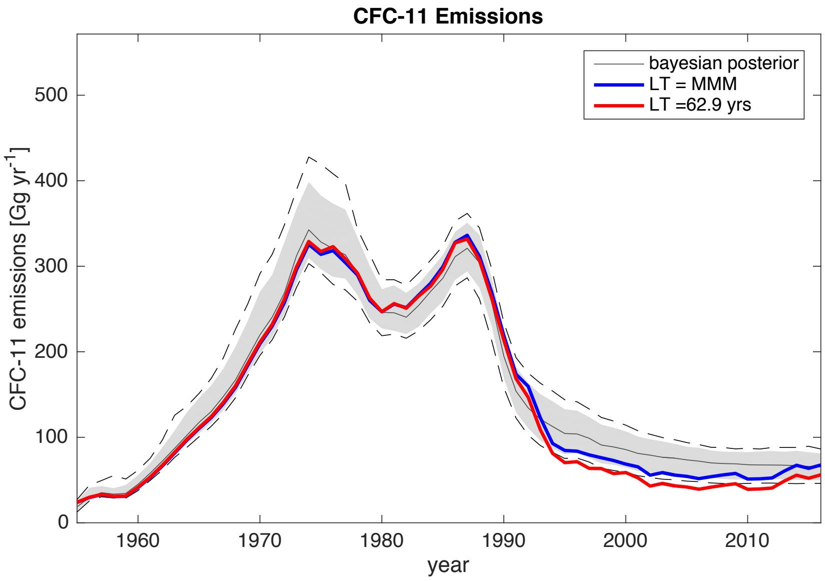

Supplementary Figure 11: Estimates of the emissions of CFC-11, analogous to Figure 3 in the main text but for the unexpected production scenario. Blue and red lines show results for observationally-derived emissions using the SPARC MMM and constant atmospheric lifetimes, respectively. The grey line indicates the mean Bayesian posterior estimate for the unexpected production scenario, the grey shaded region indicates the 95 % confidence interval and the dashed line indicates the 99% confidence interval.

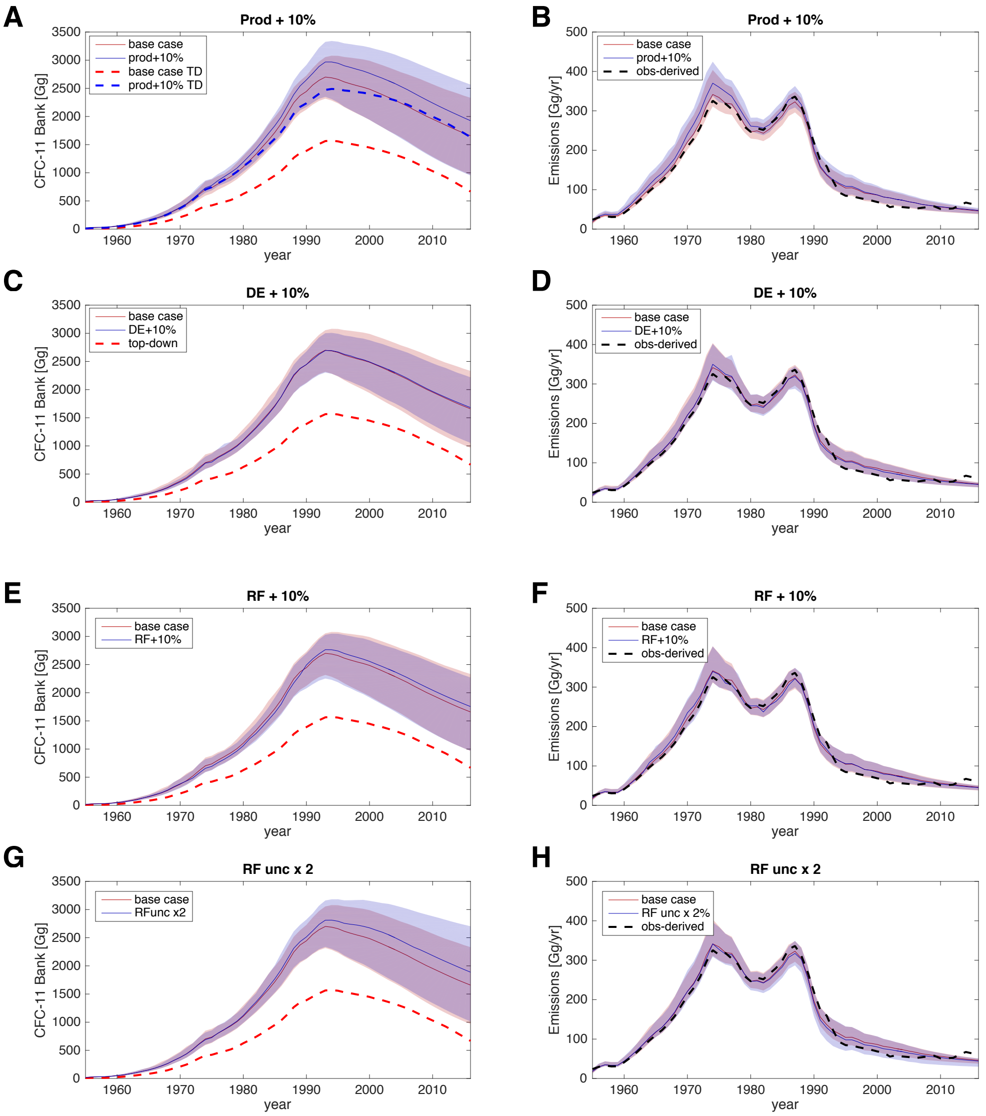

Supplementary Figure 12: Sensitivity of bank and emissions posteriors to key parameter assumptions. (a, c, e, g) CFC-11 bank posteriors. (b, d, f, h) Corresponding emissions posteriors. The 'base case' throughout refers to the SPARC MMM lifetime (red line). Shaded regions indicate 95% CI throughout. Top-down, observationally derived bank (red dashed line) for the base case and observationally derived emissions (black dashed line) are also shown. (a, b) Blue line: BPE posterior estimates with reported production values 10% higher than the base case. The blue dashed line shows the top-down bank estimates for this case. (c, d) Blue line: BPE posterior estimates where the mean of each prior distribution for direct emissions is increased by ~10%. (e, f) Blue line: BPE posterior estimates where the mean of each prior distributions for release fraction is increased by ~10%. (g, h) Blue line: BPE posterior estimates where the standard deviation in the prior distributions for release fraction on closed-cell foams is doubled (i.e. Lognormal with parameters such that $\mu = 3.66\%$ and $\sigma = 3.66\%$, see Supplementary Table 1 for comparison with base case).

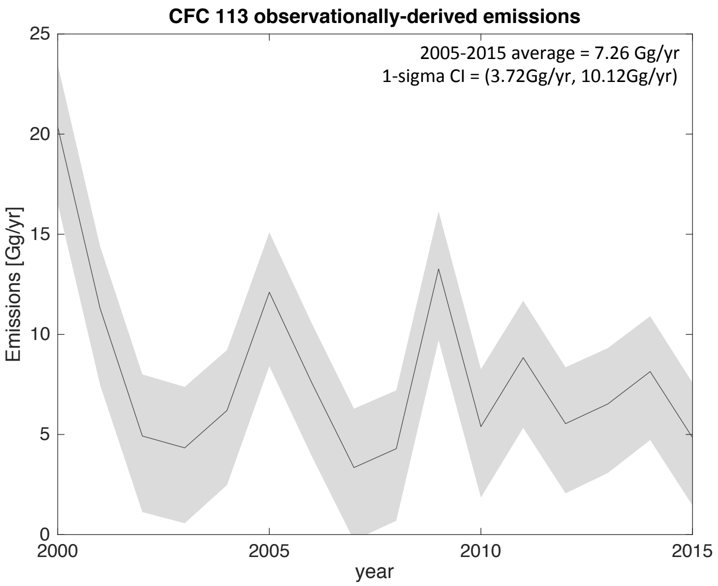

Supplementary Figure 13: Observationally derived emissions of CFC-113. The black line shows emissions estimates using a lifetime of 85 years and observed mole fractions. The shaded areas indicate the uncertainty in emissions due to a 1-sigma lifetime uncertainty of +/- 15%. The average across 2005-2015 and the +/- 1-sigma confidence interval is also shown.

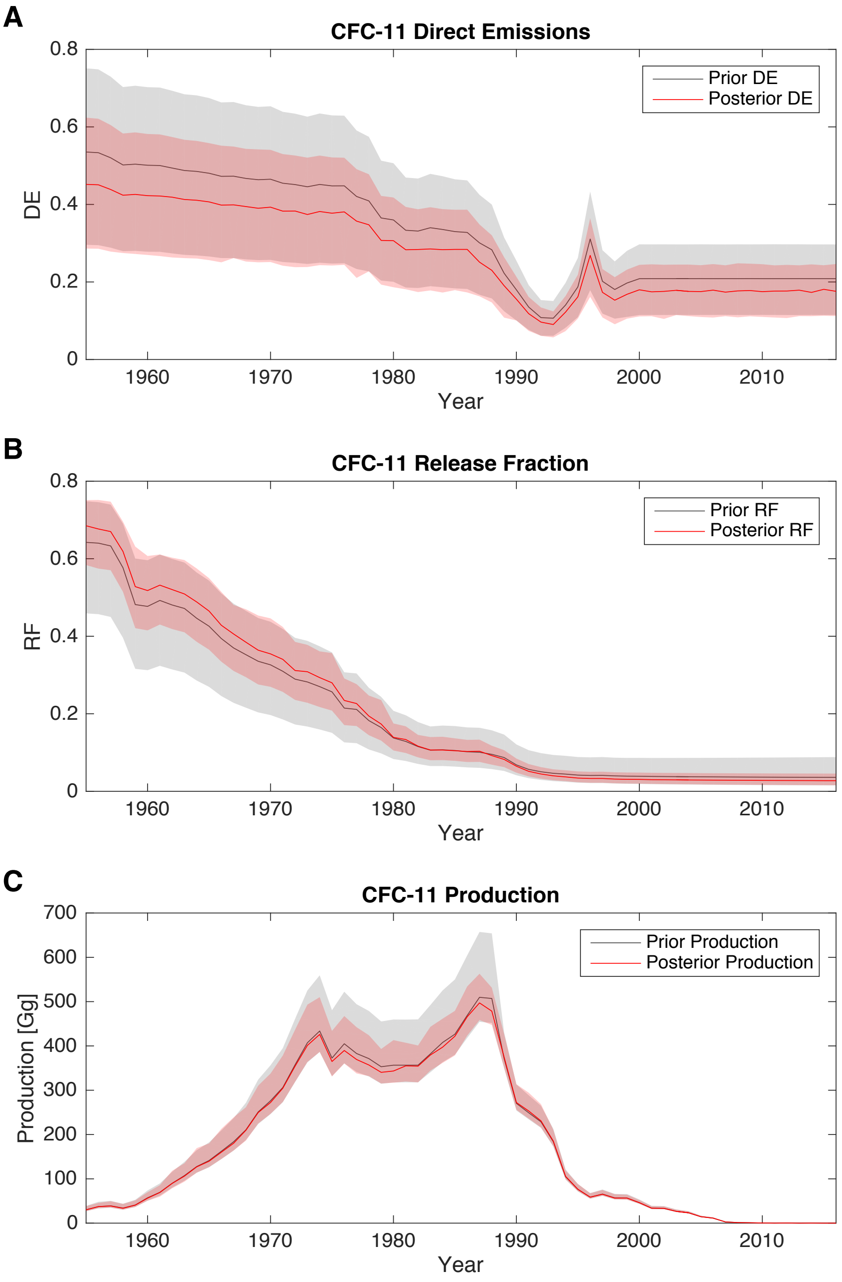

Supplementary Figure 14: Timeseries of prior (grey) and posterior (pink) 95% confidence intervals for the input parameters to the simulation model for CFC-11 for (a) Direct Emissions, (b) Release fraction and (c) production.

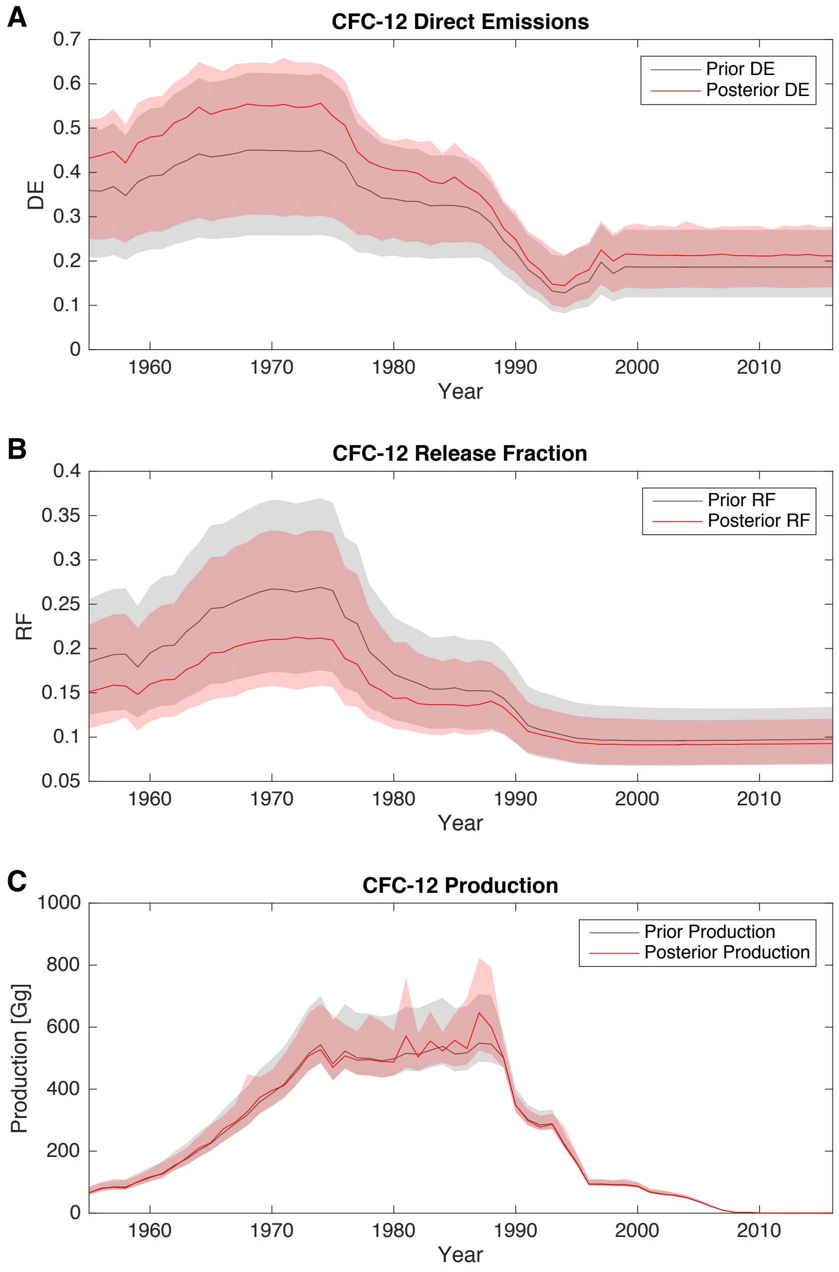

Supplementary Figure 15: As in Supplementary Figure 15 but for CFC-12

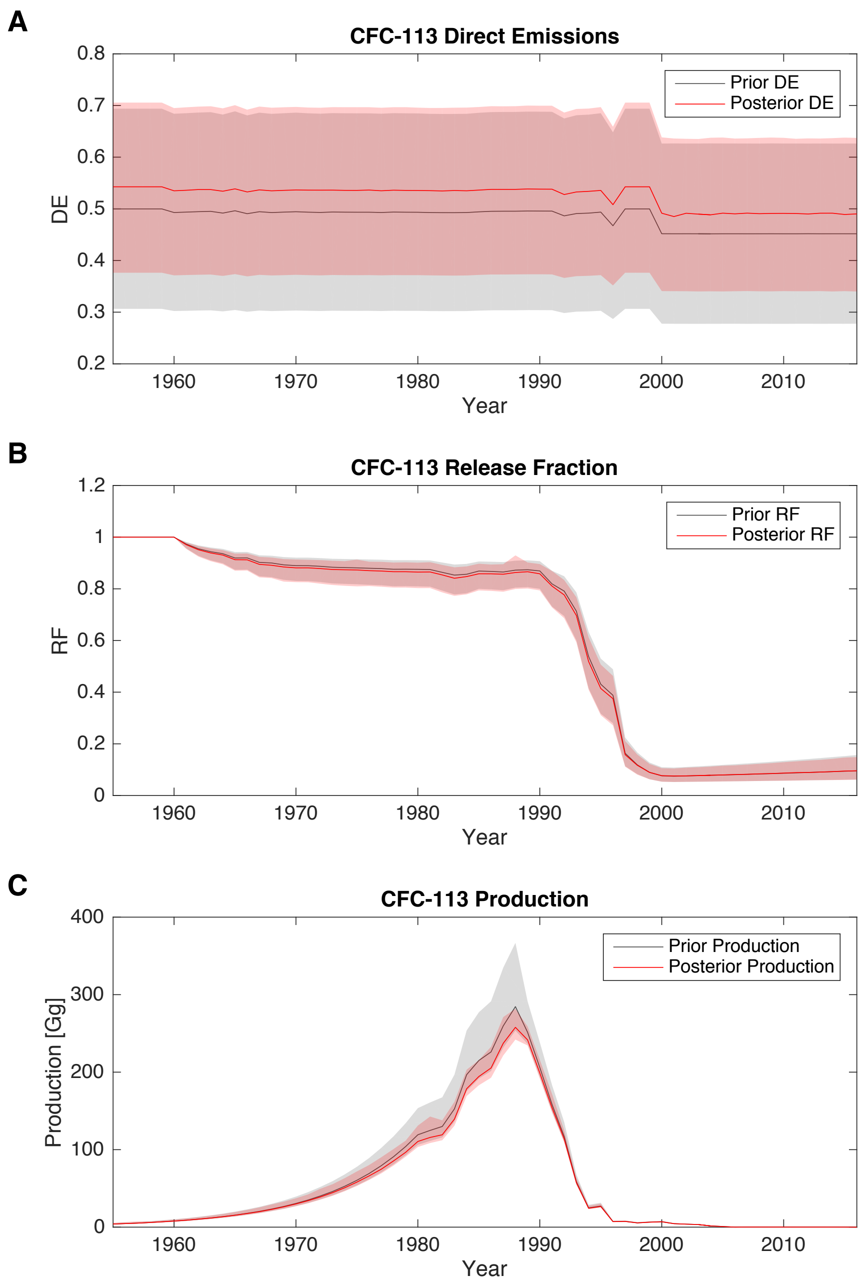

Supplementary Figure 16: As in Supplementary Figure 15 but for CFC-113

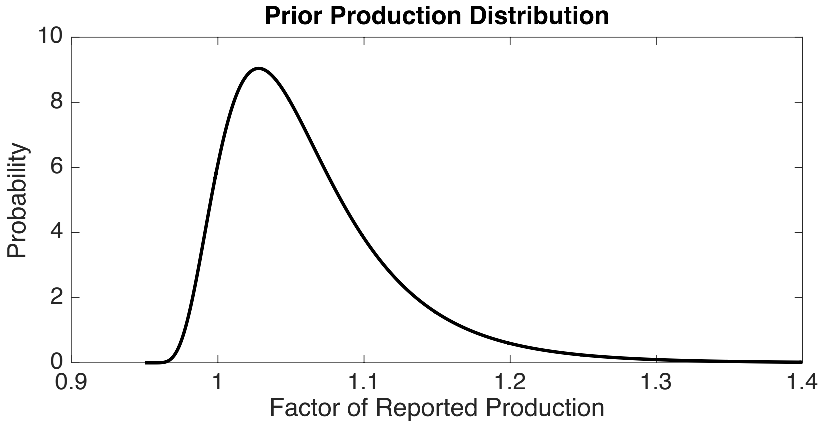

Supplementary Figure 17: Prior probability distribution for production samples.

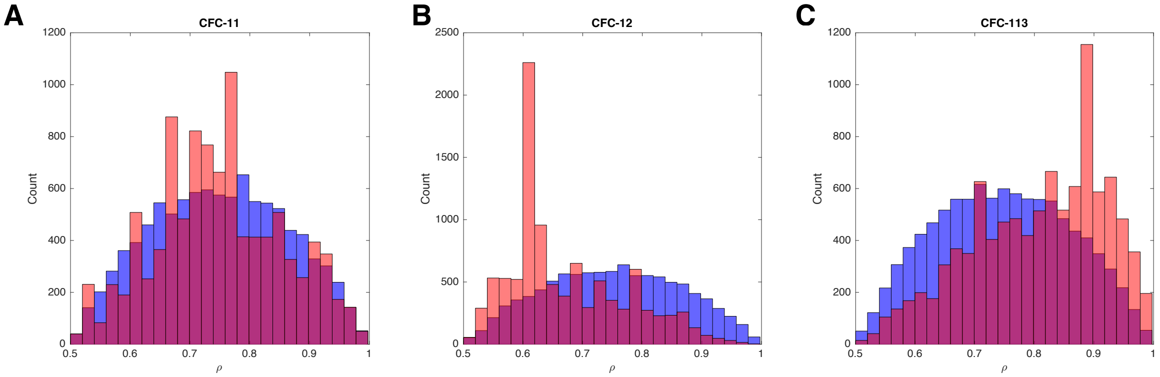

Supplementary Figure 18: Prior (blue) and posterior (pink) samples of ρ_1, the production autocorrelation term estimated in the BPE for each of the molecules run using the SPARC MMM LT scenario. Distributions are shown for (a) CFC-11, (b) CFC-12 and (c) CFC-113.

Supplementary References

1. Adcock, K. E. *et al*. Continued increase of CFC-113a (CCl3CF3) mixing ratios in the global atmosphere: Emissions, occurrence and potential sources. *Atmos. Chem. Phys.* **18,** 4737–4751 (2018).
